# The N-Terminal Residues 43 to 60 Form the Interface for Dopamine Mediated α-Synuclein Dimerisation

**DOI:** 10.1371/journal.pone.0116497

**Published:** 2015-02-13

**Authors:** Su Ling Leong, Mark G. Hinds, Andrea R. Connor, David P. Smith, Eva Illes-Toth, Chi L. L. Pham, Kevin J. Barnham, Roberto Cappai

**Affiliations:** 1 Department of Pathology, The University of Melbourne, Parkville, Victoria, 3010, Australia; 2 Bio21 Molecular Science and Biotechnology Institute, The University of Melbourne, Parkville, Melbourne, Victoria, 3010, Australia; 3 School of Chemistry, The University of Melbourne, Parkville, Melbourne, Victoria, 3010, Australia; 4 Biomedical Research Centre, Sheffield Hallam University, Sheffield, S1 1WB, United Kingdom; 5 Florey Institute of Neuroscience and Mental Health, The University of Melbourne, Parkville, Melbourne, Victoria, 3010, Australia; 6 Department of Pharmacology, The University of Melbourne, Parkville, Melbourne, Victoria, 3010, Australia; University of Padova, ITALY

## Abstract

α-synuclein (α-syn) is a major component of the intracellular inclusions called Lewy bodies, which are a key pathological feature in the brains of Parkinson’s disease patients. The neurotransmitter dopamine (DA) inhibits the fibrillisation of α-syn into amyloid, and promotes α-syn aggregation into SDS-stable soluble oligomers. While this inhibition of amyloid formation requires the oxidation of both DA and the methionines in α-syn, the molecular basis for these processes is still unclear. This study sought to define the protein sequences required for the generation of oligomers. We tested N- (α-syn residues 43–140) and C-terminally (1–95) truncated α-syn, and found that similar to full-length protein both truncated species formed soluble DA:α-syn oligomers, albeit 1–95 had a different profile. Using nuclear magnetic resonance (NMR), and the N-terminally truncated α-syn 43–140 protein, we analysed the structural characteristics of the DA:α-syn 43–140 dimer and α-syn 43–140 monomer and found the dimerisation interface encompassed residues 43 to 60. Narrowing the interface to this small region will help define the mechanism by which DA mediates the formation of SDS-stable soluble DA:α-syn oligomers.

## INTRODUCTION

Parkinson's disease (PD) is a progressive neurodegenerative disorder characterised by severe motor dysfunction, muscular rigidity and resting tremor. [1] Pathologically, PD is characterised by the presence of intracellular protein inclusions: Lewy bodies in the neuronal cell bodies and Lewy neurites in the neuronal processes. [2] A major constituent of the Lewy body and Lewy neurites is the α-synuclein (α-syn) protein. α-syn aggregates, over time, into amyloidogenic fibrils and this can be modulated by a range of factors including protein concentration, anions, pesticides, metals, neurochemicals and proteins. [3, 4] α-syn is an intrinsically disordered protein (IDP) comprising 140 amino acids. It contains three regions with distinct structural characteristics: the N-terminus encompassing residues 1–60 contains a series of imperfect repeats with a consensus motif of KTKEGV, residues 61–95 encoding the non-amyloid β component of the Alzheimer’s disease amyloid (NAC) forms the amyloidogenic core of α-syn, and the C-terminus encompassing residues 96–140 which is a highly negatively charged region. [5, 6]

The loss of dopaminergic neurons in the substantia nigra, and the subsequent depletion of dopamine (DA) in the striatum is a key feature of PD. α-syn expression can regulate DA metabolism since α-syn knockout mice have reduced striatal DA levels and an attenuated DA-dependent locomotor response to amphetamine. [7] There is an important interaction between DA and α-syn resulting in DA (and related catecholamines) inhibiting α-syn fibrillisation into amyloid [8–15] and inducing the formation of SDS-stable soluble oligomers. [8, 13, 16] The interaction of DA with α-syn results in the oxidation of all four methionine residues in α-syn, a process necessary for the formation of the soluble α-syn oligomers. [17, 18] The catechol mediated aggregation of α-syn also requires their oxidation [10, 12, 19] and can involve the interaction of the oxidised catechols with the YEMPS sequence in the C-terminal region of α-syn. [12] DA oxidation is a complex process resulting in diverse metabolites with different properties and that can either have toxic or protective effects [20]). An oxidative metabolite of DA is dopamine quinone and this forms covalent adducts with α-syn to yield soluble oligomers. [10, 16, 21] While the nature of the DA linkage to α-syn is still ill-defined, the DA:α-syn oligomers are highly stable and remain intact even in the presence of strong denaturants such as 6 M guanidine hydrochloride or urea. [22] Formation of DA:α-syn oligomers can be modulated by pH and lipids. [19] Lipids inhibit the formation of the DA-induced α-syn oligomers [23] while the DA metabolite 5,6-dihydroxylindole promotes soluble α-syn oligomer formation at physiological pH, but under alkaline conditions gives to insoluble oligomers. [19]

There is a clear difference in behaviour between α-syn and the DA:α-syn oligomer since the DA:α-syn oligomer cannot interact with lipid vesicles or cause membrane permeability while α-syn (in the absence of DA) does. [23] While DA-induced α-syn oligomers are detectable in cells [8, 14, 15], it is unclear if they represent toxic [11, 14] or non-toxic [8, 15] species. Their role in cells may relate to a proposed function in modulating energy metabolism, oxidative stress and proteostasis. [3]

Structural analysis using atomic force microscopy indicated the DA:α-syn oligomers possessed non-fibrillar morphologies. [12, 24] Electrospray-ionisation-ion mobility spectrometry-mass spectrometry (ESI-IMS-MS) [25] and small angle X-ray scattering (SAXS) [22, 26] showed DA binds to α-syn when the protein is in an extended conformation. Single-molecule photobleaching and substoichiometric fluorescent labeling determined that DA:α-syn aggregates form as two distinct species a minor assembly containing 15–19 monomers and a major assembly of 34–38 monomers. [27] However, smaller oligomeric assemblies are also present in solution since dimeric to tridecamer DA:α-syn species have been fractionated by size exclusion chromatography (SEC). [24, 26] Nuclear magnetic resonance (NMR) and molecular dynamic stimulations suggested the N-terminal imperfect repeats and glutamate 83 in the NAC domain are necessary for interactions with DA. [28] To understand the nature of the interaction between DA and α-syn we have investigated which regions of α-syn are required for the formation of the DA-mediated oligomers. We used an N-terminal truncated dimeric species of α-syn, encoding residues 43–140, for detailed structural and biophysical analysis. We identified the region between residues 43 to 60 to contain residues necessary for DA mediated dimerisation of α-syn.

## Experimental Procedures

### Production of α-syn

Full-length (1–140) human α-syn cDNA sequence was cloned into the vector pRSETB (Invitrogen, Carlsbad, CA) and expressed and purified as previously described. [17] Purified α-syn fractions were pooled, dialysed against water and lyophilised. Protein identity was confirmed by mass spectrometry. Different batches of the protein were expressed and purified during this study.

The α-syn 1–60, 1–95, 43–140, H50N, T75P constructs were made using the QuikChange Site Directed Mutagenesis system (Invitrogen, Carlsbad, CA). α-syn 1–60 was also produced synthetically using FMOC peptide synthesis (Applied Biosystems, Foster City, CA). The α-syn 43–140 construct has a Met as the first residue. All truncated and mutant α-syn proteins except α-syn 1–60 and 1–95 were expressed and purified as for wildtype α-syn above. The α-syn 1–60 and 1–95 proteins were purified by ammonium sulfate (50%) precipitation, whereby solid ammonium sulphate was added to the lysed cell mixture (v/v) and stirred overnight at 4°C. Following centrifugation at 3200 × g for 1 hour at 4°C, the supernatant discarded and the pellet resuspended in 10 mM sodium phosphate buffer pH 7.5. After boiling for 20 mins and centrifugation at 3200 × g for 1 hour at 4°C the supernatant was collected. The supernatant was desalted using a PD-10 desalting column (GE Healthcare, NSW, Australia) and then loaded on a HiTrap SP Sepharose FF cation exchange column. Bound α-syn 1–60 and 1–95 were eluted using a linear salt gradient from 0 to 1M NaCl in 20 mM Tris HCl at pH 8.5. Fractions containing α-syn 1–60 and 1–95 were pooled and lyophilised. The sample was resuspended in 100 mM ammonium bicarbonate pH 7.5 and loaded onto a Sephacryl 300 SEC column (Amersham Biosciences, Castle Hill, Australia) and eluted isocratically with 100 mM ammonium bicarbonate (pH 7.5) at a flow rate of 1mL/min. α-syn 1–60 containing fractions were pooled, dialysed and lyophilised. Protein identities were confirmed by mass spectrometry. The α-syn 43–60 peptide was synthesised using FMOC peptide synthesis (Applied Biosystems, Foster City, CA)

### Minimal media expression of α-syn

α-syn was isotopically labelled either singly (^15^N) or doubly (^15^N, ^13^C) for NMR studies. [29] An overnight culture was inoculated into 2 L of LB media. When an A_600_ of ~0.6 was achieved, the cells were pelleted by centrifugation at 3200 × g for 15 minutes at 4°C. Cells were washed twice, centrifuging after each wash with M9 minimal media to remove contaminating LB media. Cells were resuspended in M9 minimal media containing either ^15^NH_4_Cl or ^15^NH_4_Cl and ^13^C glucose and allowed to equilibrate at 37°C shaking at 220 rpm for 2 hours. Expression was induced with 1 mM IPTG overnight at 37°C. Cells were harvested by centrifugation at 3200 × g, 1 hour at 4°C. Cell pellets were stored at -20°C until lysis and purification were performed according to the method outlined above. Different batches of the protein being expressed and purified during this study.

### α-syn stock solutions

α-syn stock solutions were made up to 1mg/mL in water, and filtered through a 0.2μm pore filter to remove preformed aggregates (0.2μm Minisart RC4 filters, Sartorius, Goettingen, Germany). The concentration of stock solutions were determined by UV absorbance measurements using an extinction coefficient of 5120M^-1^ cm^-1^ at λ = 280nm.

### SDS-PAGE electrophoreses

Samples were prepared in SDS sample buffer (containing 4% SDS, 20% glycerol, 0.1% Bromophenol Blue, 50 mM Tris-HCl pH 6.8 and 10% β-mercaptoethanol) (v/v) and heated at 100°C for 10 minutes. The samples, unless otherwise stated, were separated on a 12% Bis-tris SDS-PAGE gel (Life Technologies) and the proteins visualised by silver staining according to manufacturer’s instructions (Bio Rad). Some samples were analysed on a Novex 16% Tricine SDS-PAGE gel (Life Technologies) according to the manufacturer’s instructions.

### Preparation of oxidised dopamine.

Oxidised DA was generated by incubating 2mM DA in PBS pH 7.4 at 30°C and shaking for 28 days. The reaction was then centrifuged at 200,000 × rpm for 60 min to obtain a pellet (insoluble oxidised DA corresponding to polymerised oxidised DA) and a supernatant (soluble oxidised DA) fraction. The pellet fraction was washed 3× with water and resuspended in the original volume with water. The supernatant fractions were analysed by ESI-MS and an UV-visible absorbance spectrum acquired between 200–700nm confirmed the absence of un-oxidised DA in the sample.

### Formation of DA-mediated α-syn soluble oligomers

DA mediated α-syn oligomerisation was performed as previously described [17] using 20 μM α-syn in 10 mM phosphate buffer pH 7.4 incubated with 200 μM DA at 37°C for 18 hours, unless otherwise stated. To analyse the soluble species the samples were transferred to polycarbonate tubes (7 × 20 mm polycarbonate tubes, Beckman Coulter, Fullerton, CA) and centrifuged at 100,000 x rpm for 1 hour at 4°C (TL-100 ultracentrifuge, Beckman Coulter). Supernatants were collected for further analysis.

### Purification of α-syn 43–140 oligomers for NMR analysis

Oligomerisation of α-syn for NMR analysis was performed by incubating 15mg of α-syn 43–140 at a concentration of 200 μM with 2 mM DA at 37°C on a heat block for 7 days to ensure maximal oligomerisation had occurred. After 7 days, the mixture was centrifuged 100,000 × rpm for 1 hour at 4°C and the supernatant collected. The supernatant of the crude reaction mixture was loaded onto a Superdex 200 16/60 GL SEC column (GE Healthcare, NSW, Australia). Fractions were eluted in 10 mM sodium phosphate pH7.5. Monomer and dimer-containing fractions were pooled and concentrated using an Amicon centrifugal unit with a molecular cut-off (MWCO) of 10 kDa. The monomer was concentrated using a unit with a 3 kDa MWCO. Different batches of the oligomers were generated and purified during this study.

### Circular Dichroism Spectroscopy

Circular dichroism (CD) measurements were performed on a JASCO 815 CD spectrometer (Tokyo, Japan), using the Jasco software. Far UV-CD spectra were collected between 180–250 nm at 37°C. Measurements were recorded at 50 nm/min with a 1 nm bandwidth and a 2s response time, averaging 3 accumulation scans per measurement. All spectra were background subtracted, and smoothed with the default algorithm in the Jasco software. Experiments were performed in triplicate.

### NMR spectroscopy

NMR spectra were acquired on a Bruker-Biospin Avance 800MHz spectrometer equipped with a cryogenically cooled probe. All experiments were performed at 5°C, and samples were typically 0.5mM protein in 0.5mL 10mM phosphate buffer pH 5.5 with either 10% D_2_O/90% H_2_O or 100% D_2_O as appropriate. ^1^H,^13^C-HSQC spectra were acquired on α-syn, at natural abundance. All double-resonance spectra were acquired with 2048 complex data points in the directly detected dimension and 512 complex data points in the indirect dimension.

A series of standard two-dimensional (2D) and three-dimensional (3D) spectra were recorded to obtain sequence specific assignments, these included ^1^H, ^15^N NOESY-HSQC with a mixing time of 100 ms, ^1^H, 1H NOESY with a mixing time of 300 ms, ^1^H TOCSY with a mixing time of 50 ms, ^1^H, ^15^N-HSQC, ^1^H, ^13^C-HSQC, HNCO, HN(CA)CO, HN(CO)CA, HNCA, HNCACB, CC(CO)NH, HNHA, and HNHB. [30] The WATERGATE method was used to suppress the solvent signal. [31]Spectra were referenced to internal DSS. All NMR data was processed using the software TOPSPIN (Bruker Biospin AG) and analysed using the CARA application (Rochus Keller and Kurt Wuthrich, CARA, Institute of Molecular Biology and Biophysics, ETH Zurich.CARA cara.nmr.ch). [32]

A 120 ms ^1^H,^15^N X-filtered NOESY-HSQC experiment were performed using sample prepared by incubating α-syn in a reaction containing 50% unlabelled α-syn and 50% ^13^C, ^15^N labelled α-syn with DA in a 1:10 protein to DA ratio. This reaction was mixed and incubated at 37°C for 7 days. The solution was then centrifuged 100,000 × rpm, 1 hour at 4°C and the supernatant was then separated by SEC on a Superdex 200 16/60 column (GE Healthcare, NSW, Australia) to isolate the dimer. The dimer fractions were eluted in 10 mM sodium phosphate buffer at pH 7.5 and concentrated using an Amicon centrifugal filter unit (Millipore, NSW, Australia) with a MWCO of 10 kDa to a final volume of ~500μl.

### Trypsin digestion

Sequencing grade trypsin (Promega, Madison, WI) was added to a solution containing either α-syn 43–140 monomer or α-syn 43–140 dimer in 50 mM Tris-HCl pH 7.5 at a 1:50 trypsin to protein ratio. Typically, 1mg of protein and 20 μg of trypsin was used in each experiment. The reaction was incubated at 37°C for 18 hours and analysed using reverse phase HPLC and ESI-TOF-MS.

### Electrospray-ionisation-ion mobility spectrometry-mass spectrometry (ESI-IMS-MS)

ESI-IMS-MS was performed as described. [25] All reagents were purchased from Sigma-Aldrich, UK. Protein samples for ESI-IMS-MS experiments were prepared by dissolving α-syn to a 40 μM final concentration and by diluting DA to a final concentration of 6.25 mM in aqueous 50 mM ammonium acetate pH 6.8. All spectra were acquired using a Synapt G2 HDMS instrument (Manchester, Waters, UK) by use of gold coated home-made borosilicate nano-capillaries in positive mode. Optimised instrumental settings for data acquisition were: capillary voltage of 1.70–1.90 kV, cone voltage of 50 V, source temperature of 60°C, trap collision energy of 4.0 V, transfer collision energy of 10 V, trap bias 45, backing pressure of 3.1 mbar. IMS separations were performed at T-wave velocities of Trap:311, IMS:800 and Transfer:200 m/s and T-wave amplitudes of 4–15 V using 3.6 mbar pressure of nitrogen gas maintained by a 90 mL/min gas flow. Mass calibration was carried out by an infusion of CsI cluster ions, and arrival time distributions were determined by using the Mass Lynx v4.1 software (Waters, UK).

### Experimental replicates

Aggregation and spectroscopy experiments were repeated at least three separate times and were performed with different batches of expressed and purified protein and different batches of purified oligomers.

## Results

### N-terminally truncated α-syn 43–140 forms DA-mediated soluble oligomers

**Fig 1:**
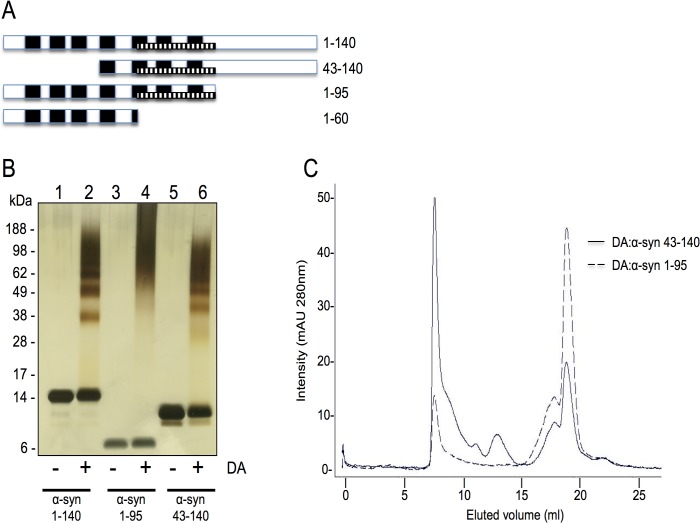
Oligomerisation of α-syn on treatment with DA. **A**. Schematic of the different α-syn constructs used in this study. The black bars represent the imperfect repeats (residues 10–16, 21–27, 32–37, 43–49, 57–63, 80–86). NAC region (stippled bar, residues 60–95). **B**. Silver stain SDS-PAGE gel of truncated α-syn incubated in the presence or absence of DA. Lane 1: α-syn 1–140. Lane 2: α-syn 1–140 + DA. Lane 3: α-syn 1–95. Lane 4: α-syn 1–95 + DA. Lane 5: α-syn 43–140. Lane 6: α-syn 43–140 + DA. The α-syn to DA ratio was 1:10. **C**. Size exclusion chromatography of DA:α-syn 43–140 and DA:α-syn 1–95 oligomers. 200 μM α-syn was incubated with 2 mM DA for 7 days. The reaction was centrifuged at 100,000 rpm, 1 hour, 4°C and then analysed on a Superdex 200 10/300GL column using 10 mM sodium phosphate pH 7.5 buffer with a flow rate of 0.5 mL/min. Proteins were detected at A280nm.

**Fig 2:**
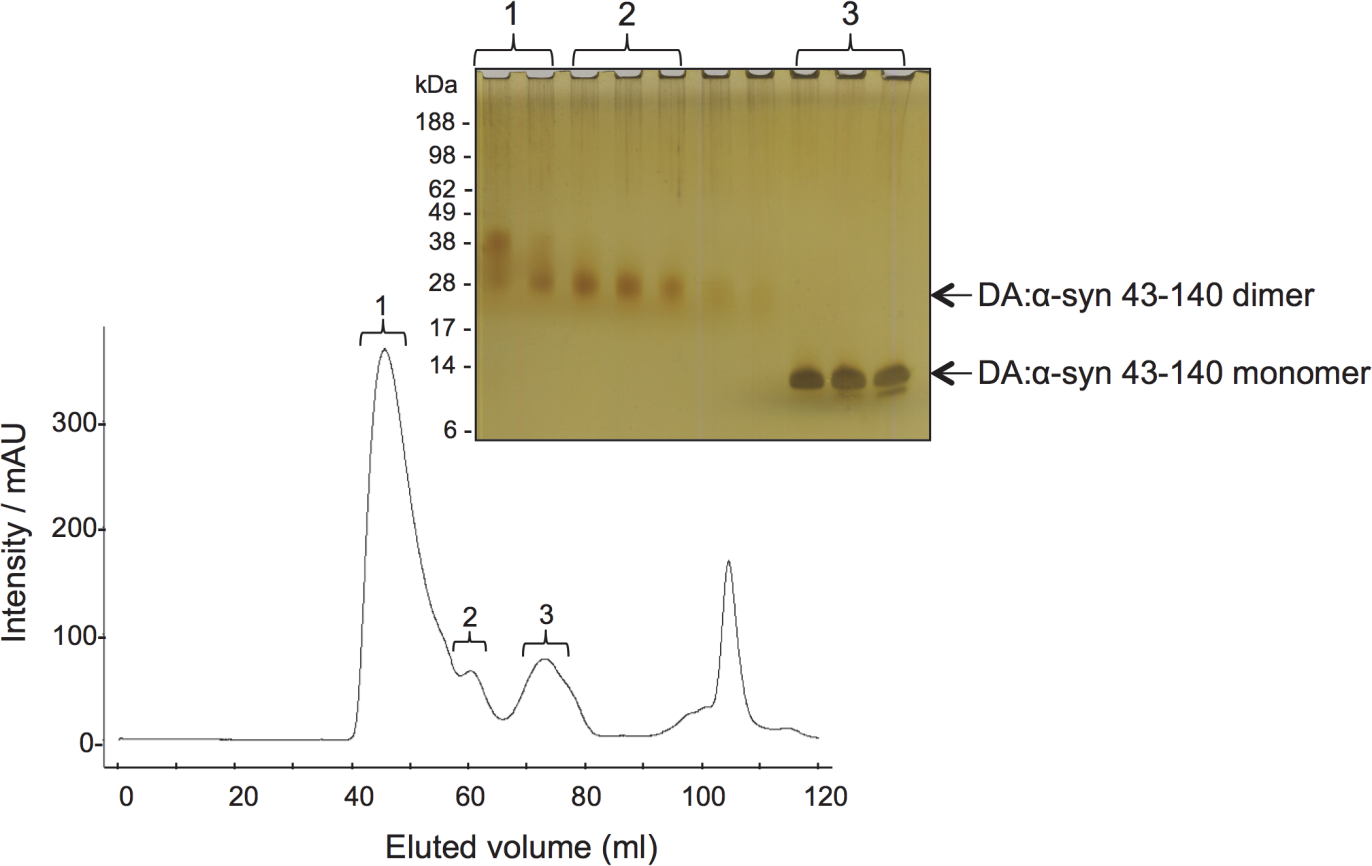
Purification of DA:α-syn 43–140 soluble oligomers. Chromatogram of size exclusion chromatography of DA:α-syn 43–140 induced oligomers on a Superdex 200 preparation grade column using 10 mM sodium phosphate pH 7.5 buffer with a flow rate of 1 mL/min. Proteins were detected at A280nm. Inset shows silver stain SDS-PAGE of samples from the selected fractions (as denoted).

To define which regions of α-syn are required for the formation of the DA-mediated soluble oligomers, we tested N- and C-terminal truncated proteins (Fig. 1A). α-syn 43–140 lacks the first three imperfect repeats of the consensus motif KTKEGVT, with the truncation occurring at the break between the two α-helices in the N-terminus. [33] In α-syn 1–95 the C-terminus was deleted just after the end of the hydrophobic NAC fragment. α-syn 1–95 or α-syn 43–140 (20 μM) were incubated with DA (200 μM) at 37°C for 18 hours, then centrifuged at 100,000 rpm for 1 hour at 4°C and the supernatants recovered and analysed by SDS-PAGE and silver staining. Both α-syn 1–95 and α-syn 43–140 formed DA-mediated soluble oligomeric species (Fig. 1B). α-syn 43–140 species that have been reacted with DA are termed DA:α-syn, The profile for α-syn 43–140 was similar to full length α-syn, while α-syn 1–95 generated a smear like pattern on SDS-PAGE following incubation with DA (Fig. 1B). The band migrating with the 28 kDa marker is the DA:α-syn 43–140 dimer, and this was subsequently confirmed by SEC (Fig. 2). We also analysed DA:α-syn 1–95 and DA:α-syn 43–140 by SEC and the migration properties of these species demonstrated DA:α-syn 1–95 and DA:α-syn 43–140 had distinct elution profiles. The DA polymers and salts eluted with the two peaks seen in the 15–20 mL fractions. In the SEC chromatograms DA:α-syn 43–140 displayed multiple bands between 5–15 mL while DA:α-syn 1–95 showed only a single major peak and lacked distinct species in the region between 10–15 mL in the elution profile (Fig. 1C). These data are consistent with the SDS-PAGE results where DA:α-syn 43–140 displayed distinct oligomeric species while DA:α-syn 1–95 migrated as a smear without intermediate lower mass oligomeric species. We can conclude that while the N- and C-terminal sequences are not critical for the formation of DA-mediated soluble oligomers the C-terminus can influence the oligomeric profile and their behaviour. The data suggests the essential region required for formation of soluble oligomers lies in the region 43–95.

The generation of DA mediated α-syn oligomerisation requires oxidation of DA to occur [10] and it is the DA oxidative intermediates, such as dihydroxyphenylacetic acid (DOPAC) [8], dopaminochrome [12] and DHI [19] that have been suggested to play important roles in promoting oligomerisation and preventing fibril formation. Moreover, the presence of anti-oxidants can prevent DA-mediated oligomer formation. [10, 19] We tested the ability of oxidised DA to promote oligomerisation of the truncated proteins. Oxidised DA was produced by incubating DA for 28 days in air, and the reaction was then centrifuged to obtain a pellet (corresponding to insoluble polymerised oxidised DA) and a supernatant (soluble oxidised DA) fraction. The supernatant fractions were analysed by ESI-MS and an UV-visible absorbance spectrum acquired between 200–700nm to confirm the absence of un-oxidised DA in the sample (data not shown). The truncated α-syn proteins were incubated with either the pellet or supernatant oxidised DA fractions, or with freshly prepared DA and then analysed by SDS-PAGE and silver staining (S1 Fig.). The soluble pre-oxidised DA was a much less effective in promoting α-syn oligomerisation, while the insoluble pre-oxidised DA fraction was inactive. This indicates that fully oxidized DA is significantly less effective in causing DA-mediated α-syn oligomerisation and therefore we conclude the oxidation reaction and formation of oxidative intermediates is required to drive this form of α-syn oligomerisation.

### The DA:α-syn 43–140 dimer is predominantly random coil, even in the presence of SDS

To study the biophysical properties of the DA:α-syn 43–140 species we reacted α-syn 43–140 with DA and the dimer and monomer were purified using SEC and their identity confirmed by SDS-PAGE (Fig. 2) and ESI-Q-TOF mass spectrometry. The monomeric DA:α-syn 43–140 species isolated from the reaction mixture (Fig. 2, peak 3) had a mass of 10,372 Da a value consistent with oxidation of the three methionine residues (expected mass 10,372 Da) [17] (there are only two Mets in the native α-syn sequence, the N-terminal methionine is a plasmid-derived residue). There was no evidence for a DA adduct on the DA:α-syn 43–140 monomer under the conditions employed. The DA:α-syn 43–140 dimer (Fig. 2 peak 2) was analysed by SELDI mass spectrometry and it had a mass of 20,855 Da. This mass does not correspond to what is expected for an oxidised α-syn 43–140 dimer (20,744 Da), and we could not assign the additional 111 Da to a specific modification or adduct indicating it’s not a “standard” modification. Fraction 1 also showed the presence of a higher molecular weight species that may be a trimer (Fig. 2, left most lane).

**Fig 3:**
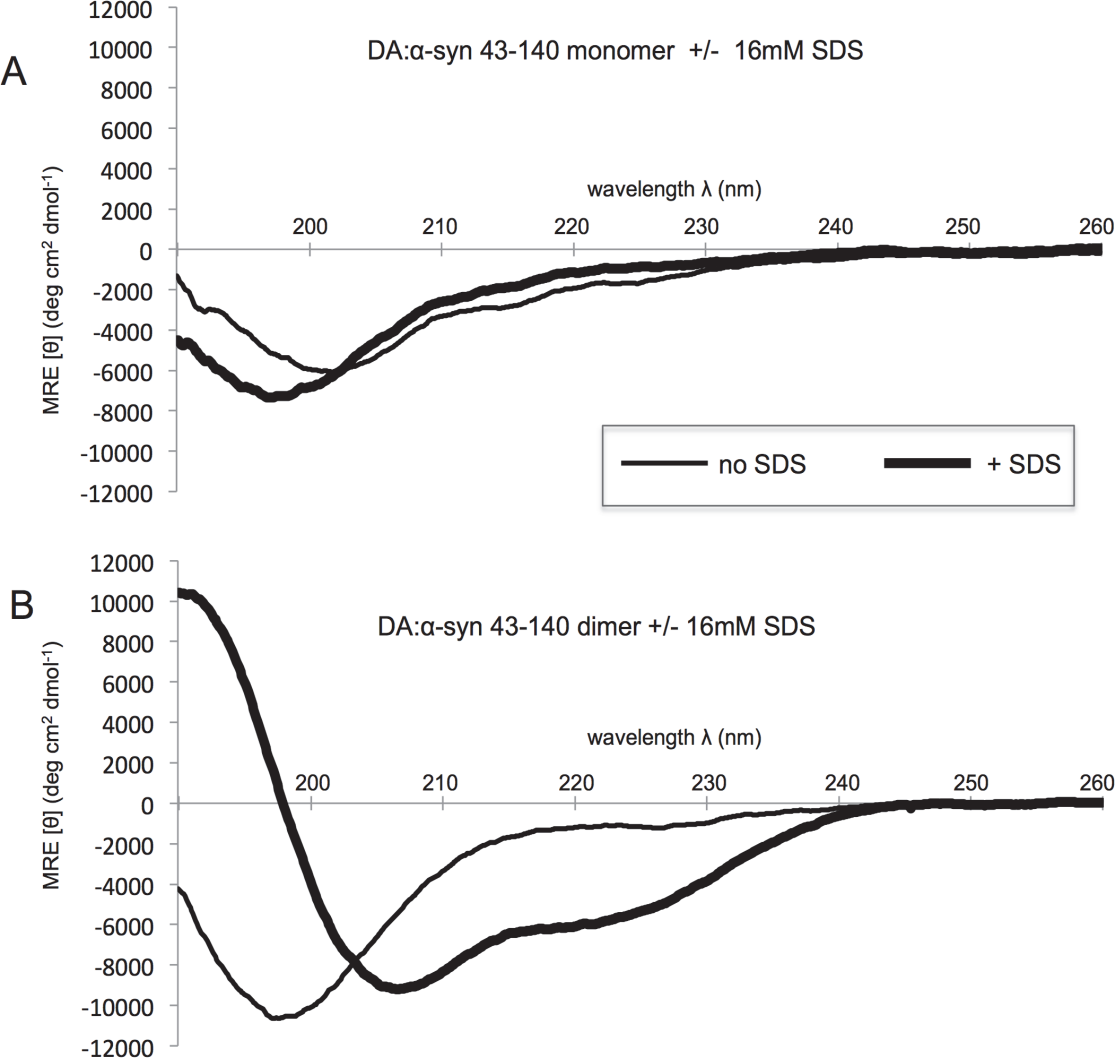
SDS-dependent conformational transition in DA:α-syn 43–140 monomer, but not DA:α-syn 43–140 dimer. Far UV CD spectrum of 15 μM DA:α-syn 43–140 monomer or DA:α-syn 43–140 dimer in 10 mM sodium phosphate buffer, pH 7.5. **A**. DA:α-syn 43–140 monomer minus (thin line) or plus 16 mM SDS (thick line). **B**. DA:α-syn 43–140 dimer minus (thin line) or plus 16 mM SDS (thick line). The CD spectrum shows the monomer and dimer in the absence of SDS have an intensity at λ = 200 nm is indicative of random coil structure. [58] In the presence of SDS, the α-syn 43–140 monomer, but not the α-syn 43–140 dimer, showed a double minima at 208 and 220 nm with a maximum at 190nm are indicative of α-helical content. [59]

The DA:α-syn 43–140 dimer was characterised by far-UV CD spectroscopy and gave a single minima at λ = 200 nm that is indicative of a predominately random coil structure. A similar spectrum was observed for the DA:α-syn 43–140 monomer (Fig. 3A). Therefore, generation of the DA:α-syn 43–140 dimer does not promote major secondary structure formation. In the presence of SDS α-syn undergoes a conformational change from random coil to α-helix, with the SDS-binding region reported to be in the N-terminus. [23, 34] The addition of SDS to the DA:α-syn 43–140 monomer resulted in a distinctive 208/220 nm double minima, indicative of α-helical structure (Fig. 3B). Therefore, the truncated N-terminus in 43–140 can still interact with SDS and undergo a structural change, supporting the validity of the construct. In the absence of SDS, there was a reduction of the negative minimum at 200 nm for DA:α-syn 43–140 dimer (compare with the monomeric species) indicating a decrease in random coil content. This observation is consistent with that of full length DA:α-syn oligomers. [26] The was no significant difference in the secondary conformation of DA:α-syn 43–140 dimer in the absence and presence od SDS (Fig. 3B). This result indicates the dimeric species is distinct from the monomer species and has a reduced propensity to interact and/or be modified by SDS, and this behaviour is consistent with the full-length 1–140 DA:α-syn dimer which can not interact with lipid vesicles. [23]

### The DA:α-syn 43–140 dimer is highly stable

**Fig 4:**
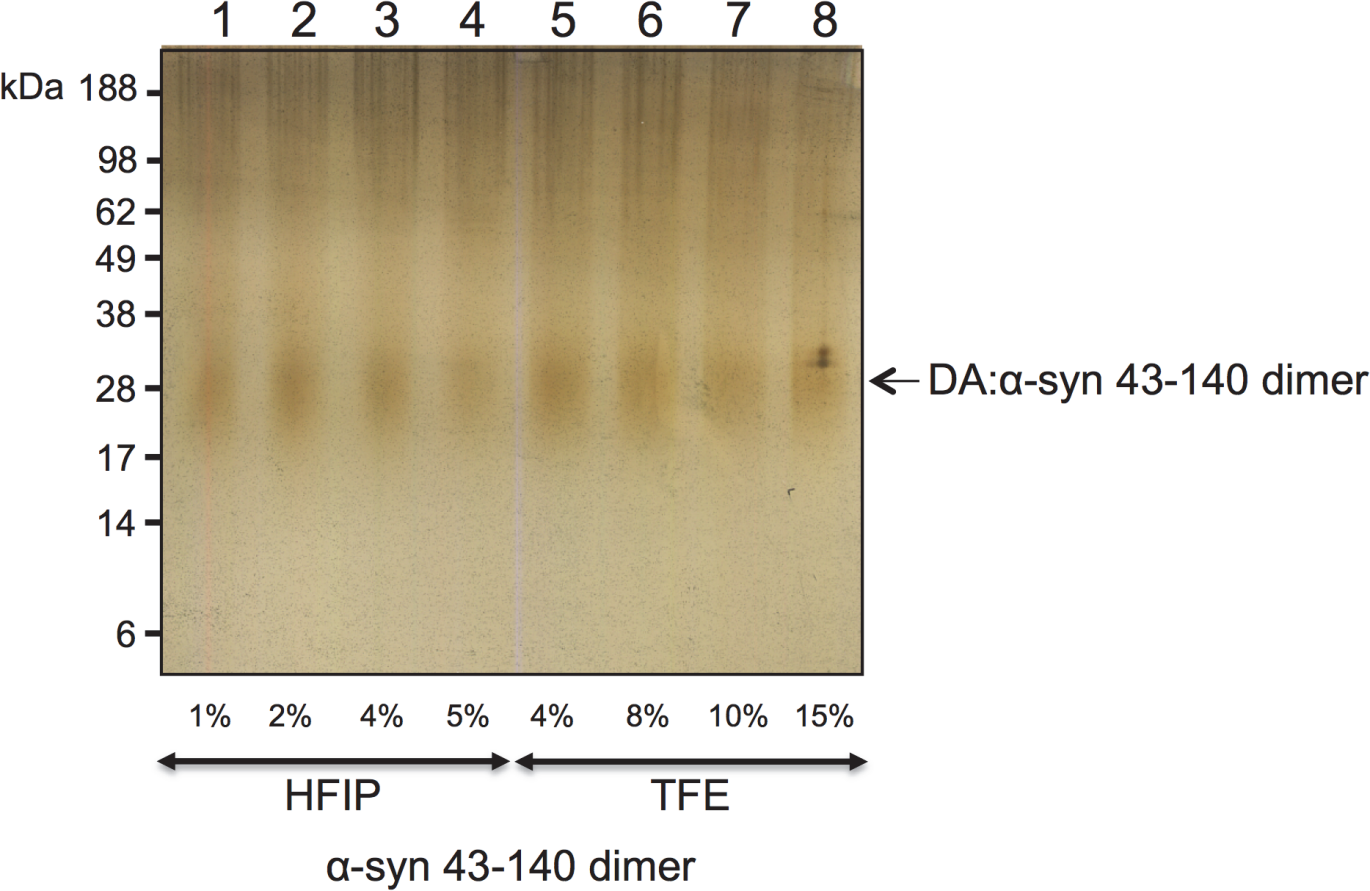
Stability of DA:α-syn 43–140 dimer exposed to denaturants. Silver stain SDS-PAGE gel of DA:α-syn 43–140 dimer incubated with different amounts of denaturants HFIP or TFE. The dimers did not dissociate under any of the conditions tested, suggesting a covalent cross-link. **Lane 1:** DA:α-syn 43–140 dimer + 1% HFIP. **Lane 2:** DA:α-syn 43–140 dimer + 2% HFIP. **Lane 3:** DA:α-syn 43–140 dimer + 4% HFIP. **Lane 4:** DA:α-syn 43–140 dimer + 5% HFIP. **Lane 5:** DA:α-syn 43–140 dimer + 4% TFE. **Lane 6:** DA:α-syn 43–140 dimer + 8% TFE. **Lane 7:** DA:α-syn 43–140 dimer + 10% TFE. **Lane 8:** DA:α-syn 43–140 dimer + 15% TFE. Arrow indicates the DA:α-syn 43–140 dimer band.

To investigate the stability of the DA:α-syn 43–140 dimer, we exposed it to trifluoroethanol (TFE) and hexafluoroisopropanol (HFIP), which are known to dissociate oligomers to their monomeric species. [35, 36] The DA:α-syn 43–140 dimer was incubated with HFIP (1%, 2%, 4%, 5%) and TFE (4%, 8%, 10%, 15%) for 18 hours at 37°C and then centrifuged at 100,000 rpm (1 hour, 4°C) to isolate the soluble supernatant fraction. The supernatant was analysed by SDS-PAGE and silver staining. The DA:α-syn 43–140 dimers did not dissociate under any of these conditions (Fig. 4). Therefore, the DA:α-syn 43–140 dimers are highly stable and resistant to conventional dissociation methods.

### α-syn 43–140 binds three DA ligands in the extended state conformation

**Fig 5:**
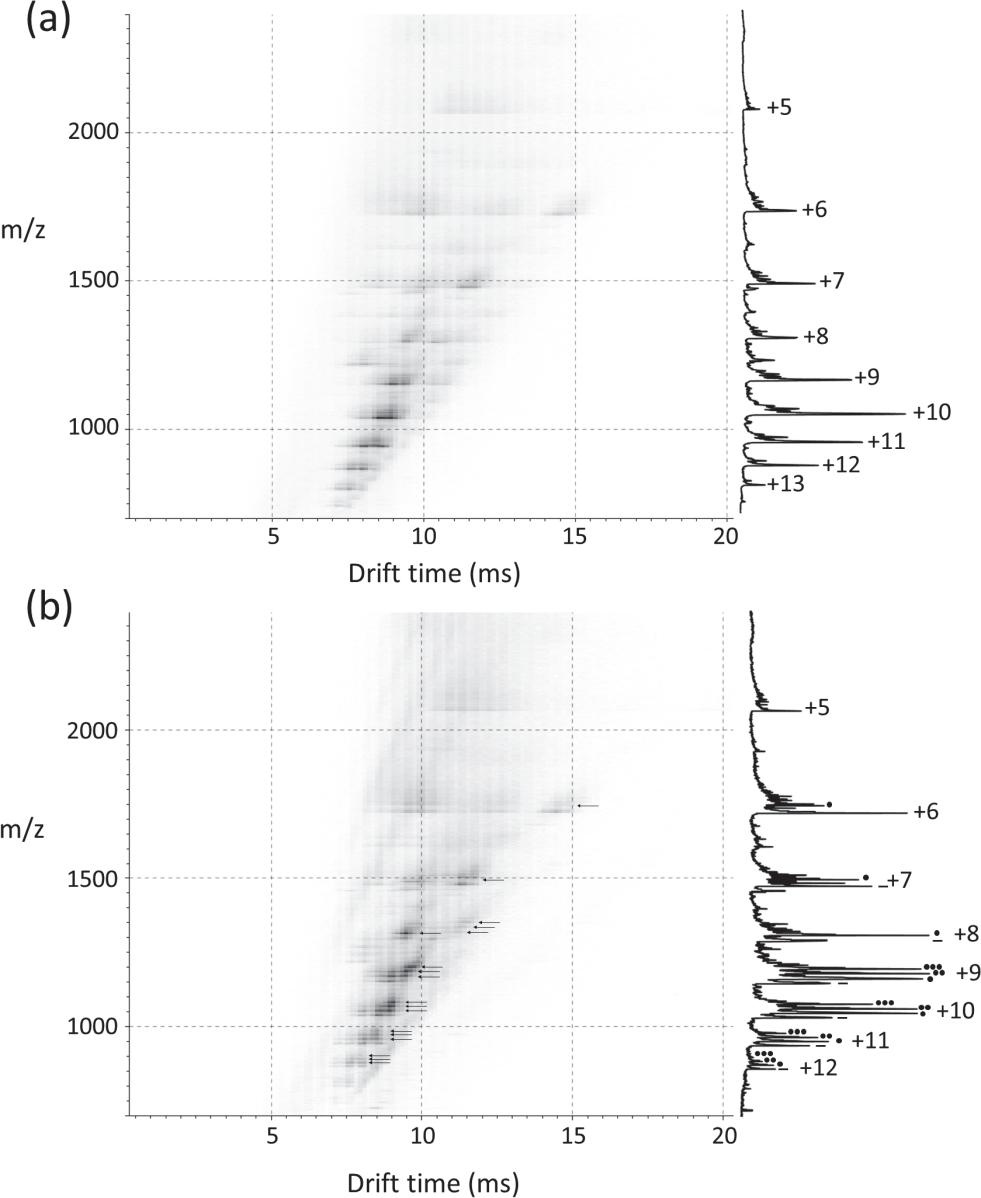
Driftscope plots of α-syn 43–140 with or without further addition of DA. The x axis represents drift time (msec), on the y axis *m/z* is shown; peak intensity is displayed on a square root scale. Corresponding mass spectrum for each acquisition is overlaid on the right of each Driftscope plot, indicating charge states and the presence or absence of the DA ligands is by the number of black dots. **A**. 40 μM α-syn 43–140 in the absence of DA. **B**. 40 μM α-syn 43–140 in the presence of DA. The asterisks refer to the number of DA molecules bound.

To further analyse the conformational properties of the DA:α-syn 43–140 protein we employed electrospray ionisation—ion mobility—mass spectrometry (ESI-IMS-MS). This method allows the gross conformation state and non-covalent interactions of a protein to be determined. [37, 38] The charge state distribution observed post-ionisation reflects the solution state of the protein in question. [39, 40] Folded or conformationally collapsed proteins typically obtain narrow charge state distributions with shortened drift times, whereas unfolded or extended proteins obtain wide charge state distributions with longer drift times. [41] Under neutral conditions full length α-syn can populate multiple extended conformational states (charge state ions +8 to +18) as well as a sub population of more compact conformational states (charge state ions +6 to +8). In the presence of DA, three DA ligands bind exclusively to a highly extended state (charge state ions +11 to +17) while the +10 charge state ion bound only a single DA ligand. The subsequent oxidised form of the protein is only observed on incubation. [25] ESI-IMS-MS analysis of monomeric α-syn 43–140 in the presence or absence of DA also populated both extended and compact conformations under neutral conditions similar to the full length protein (Fig. 5A). The extended state displayed a wide charge state distribution (+13 to +6 charge state ions) with extended drift times, whereas the compact state displayed a narrow charge state distribution (+7 to +5 charge state ions) with shortened drift times. The +7 and +6 charge state ions are co-populated by both conformational series with the most extended states being observed at the longer drift times. Similar to full length α-syn in the presence of DA, the highly extended charge state ions +13 to +8 of α-syn 43–140 also bound up to three DA ligands and the less extended charge state ions (+8 to +6) bound a single DA (Fig. 5B, arrows). The most compact states populated at the +7 to +5 charge state ions remained ligand free. The behaviour of α-syn 43–140 mirrored that previously observed for full-length α-syn in that the three DA ligands bind to the high charge state ions of the extended conformation without the requirement to first oxidise the Met residues. [25] The dimeric species was not amenable to ESI-IMS-MS.

### NMR analysis of the DA:α-syn 43–140 monomer and dimer

To analyse the behaviour and structure of the DA:α-syn 43–140 oligomers in solution we studied DA:α-syn 43–140 using NMR. This protein construct was chosen to reduce the spectral complexity arising from the presence of multiple repeat sequence motifs in α-syn and the resultant chemical shift redundancy.

**Fig 6:**
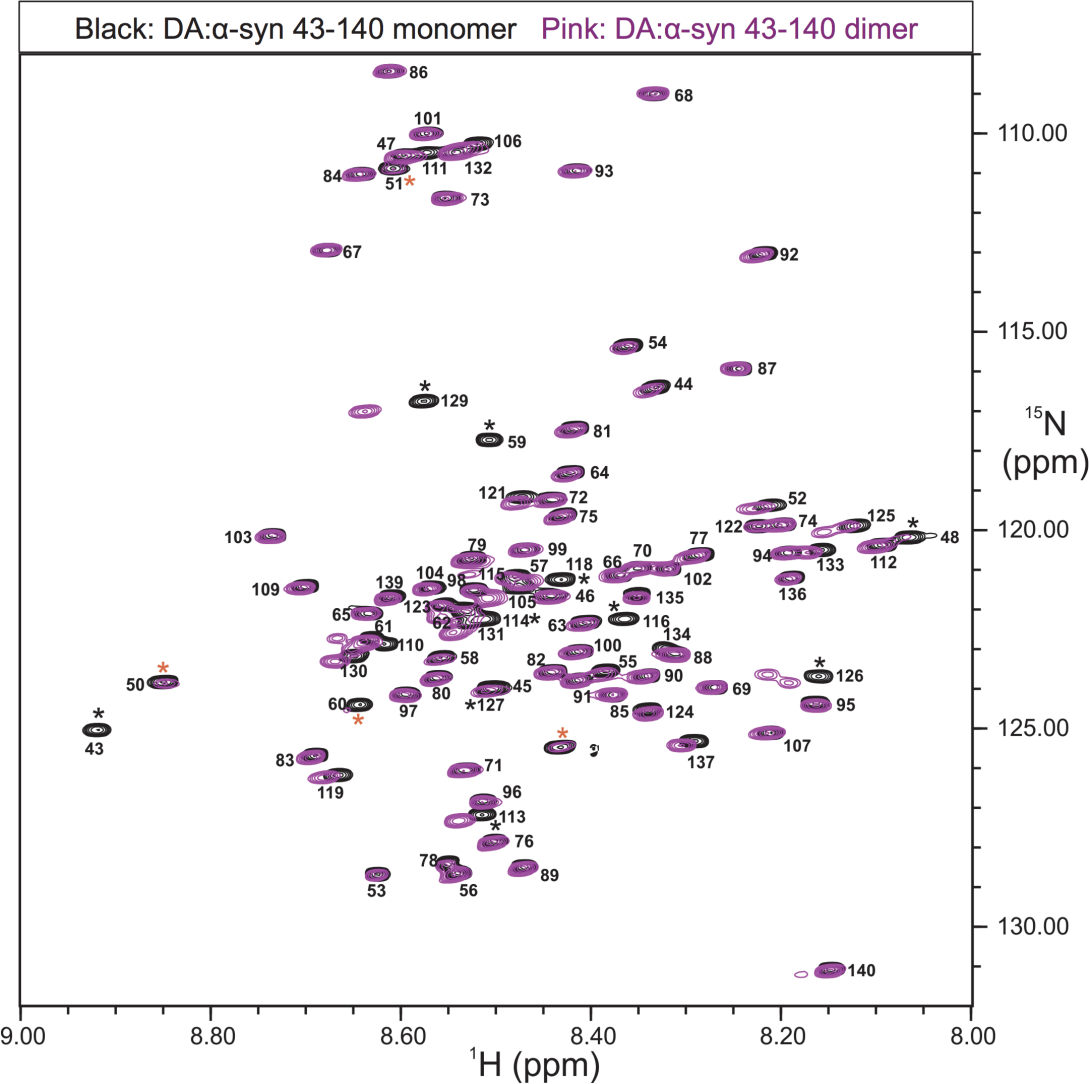
Comparison of ^1^H, ^15^N-HSQC spectra acquired on DA:α-syn 43–140 monomer and α-DA:syn 43–140 dimer showing the characteristics expected for an intrinsically disorder protein. The proton-nitrogen correlation spectrum (HSQC) of 0.5 mM DA:α-syn 43–140 monomer or DA:α-syn 43–140 dimer in 10 mM sodium phosphate buffer, pH5.5. Spectra were acquired at 5°C and 800MHz. The HSQC spectrum of DA:α-syn 43–140 monomer is depicted in black and the DA:α-syn 43–140 dimer in pink. Amide resonance assignments are indicated for the DA:α-syn 43–140 monomer as sequence position and the poor chemical shift dispersion indicates little if any secondary structure is present. * indicate residues of interest, those colored orange indicate resonances with exchange broadening (see the text).

Sequence specific NMR resonance assignments were determined for DA:α-syn 43–140 monomer and dimer using a set of spectra acquired on either singly (^15^N) or doubly (^15^N, ^13^C) labelled protein (refer Methods). A proton nitrogen correlation spectrum (^1^H, ^15^N-HSQC) of the DA:α-syn 43–140 monomer was recorded at pH 5.5 to ensure favourable amide proton exchange rates. The ^1^H, ^15^N-HSQC spectrum of the DA:α-syn 43–140 monomer displayed the characteristic narrow chemical shift dispersion in the amide ^1^H resonances for an IDP [42], of about 1 ppm (Fig. 6). [43, 44] The resonance assignments were consistent with those reported previously for α-syn. [45, 46] Similar to α-syn 43–140, the ^1^H, ^15^N HSQC spectrum of DA:α-syn 43–140 dimer also showed the characteristic lack of chemical shift dispersion for the amide ^1^H resonances expected for an IDP (Fig. 6). This indicates that like the monomer the DA:α-syn 43–140 dimer is essentially unstructured in solution and this finding is consistent with the CD data (Fig. 3).

**Fig 7:**
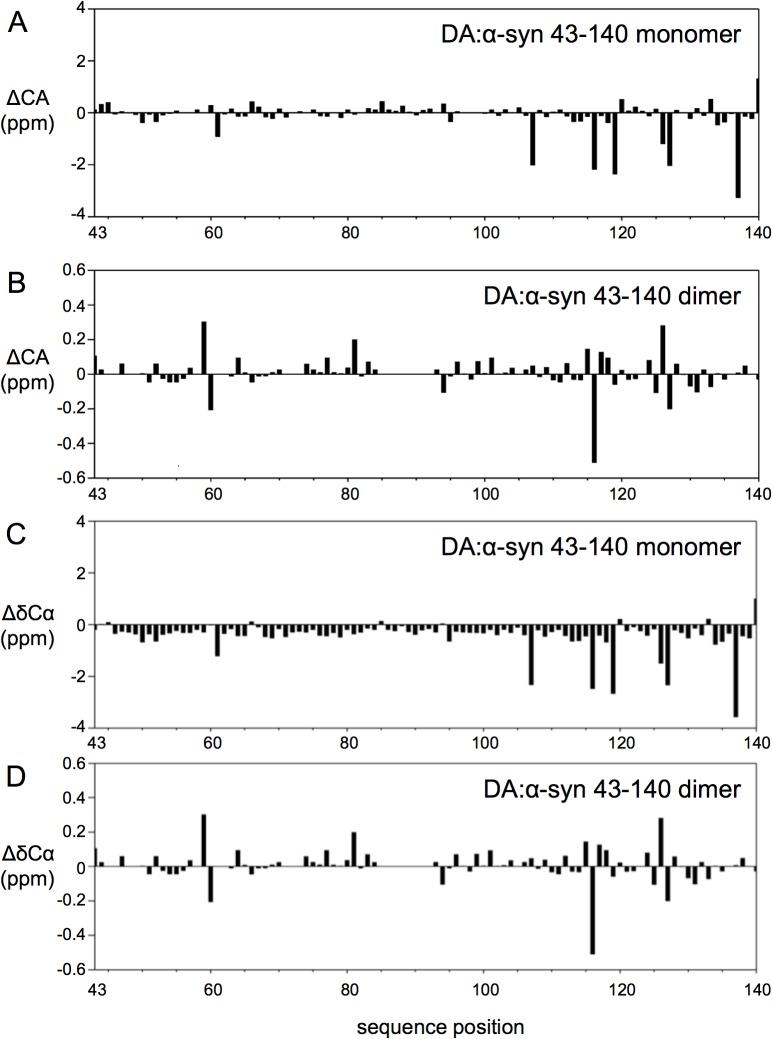
Chemical shift deviations between DA:α-syn 43–140 monomer and DA:α-syn 43–140 dimer. The Cα resonances (Panels A and B) of the DA:α-syn 43–140 dimer do not differ significantly from those of the DA:α-syn 43–140 monomer. **A**. Cα chemical shift deviations from random coil values predict that the DA:α-syn 43–140 monomer is largely unstructured. **B**. Plot of chemical shift difference for Cα chemical shifts between DA:α-syn 43–140 monomer and DA:α-syn 43–140 dimer. The chemical shift deviation is plotted in ppm as a function of residue position in DA:α-syn 43–140 monomer. **C**. Plot of difference in Cα resonance position (ΔδCα (ppm)) from random coil values [60] for DA:α-syn 43–140 monomer. The largest differences arise from residues preceding a proline (A107, M116, D119, M127, E138). **D**. Plot of difference in Cα resonance positions between DA:α-syn 43–140 monomer and DA:α-syn 43–140 dimer (δ_dimer_—δ_monomer_). The absence of significant chemical shift differences between the DA:α-syn 43–140 dimer and DA:α-syn 43–140 monomer indicates their structural similarity. The largest differences are localised to residues near the methionine residues (M116, M127) that are likely to be oxidised under the conditions of DA-mediated α-syn 43–140 dimer formation.

Resonance positions in NMR are highly sensitive to nuclear electronic environment and changes in chemical shift positions can indicate changes in conformation, for example, the Cα chemical shift in proteins, is dependent on the φ,ψ backbone torsion angles and indicative of secondary structure. [47] Monitoring changes in chemical shifts may indicate conformational change. Overlaying the ^1^H, ^15^N HSQC spectra of DA:α-syn 43–140 dimer and monomer identified 13 resonances that were shifted or broadened in the spectra of the DA:α-syn 43–140 dimer compared to those in the monomer, while the rest of the resonances were less affected. The residues with the more significant chemical amide chemical shift differences were: T43, V48, V49, H50, G51, T59, K60, L113, E114, M116, V118, E126, M127, and S129 (indicated with * in Fig. 6). Resonances of residues V48, V49, H50, K60 (indicated with an orange * in Fig. 6) were weakened in intensity in the spectra of the DA:α-syn 43–140 dimer but not shifted significantly from the position in the monomer and suggest an exchange broadening process is occurring for these residues in the dimer. The near-identical chemical shifts observed for the majority of amide resonances between monomer and dimer is consistent with a high degree of structural similarity between them. This suggests a very limited intermolecular interface for DA:α-syn 43–140 dimer that is confined to only the few residues involved in a putative cross-link. Further analysis of chemical shift for the Hα, C’ and Cα resonances, that are sensitive to backbone conformation [47], of the DA:α-syn 43–140 dimer showed they only had minor deviations from their expected random coil positions for, again further confirmation of the lack of regular secondary structure observed for this protein (Fig. 7A and 7B). The difference between the Cα chemical shifts of the DA:α-syn 43–140 monomer and dimer were plotted to determine any potential changes in conformation following dimer formation. Little difference was observed in the Cα chemical shift positions between the monomer and dimer, indicating that the formation of the dimer maintains the overall random coil conformation of the monomer (Fig. 7C and 7D). The largest differences in chemical shift are located in the vicinity of the methionines (M116, M127) and likely due to the methionine oxidation. [17]

### Trypsin digestion of the DA:α-syn 43–140 dimer

**Fig 8:**
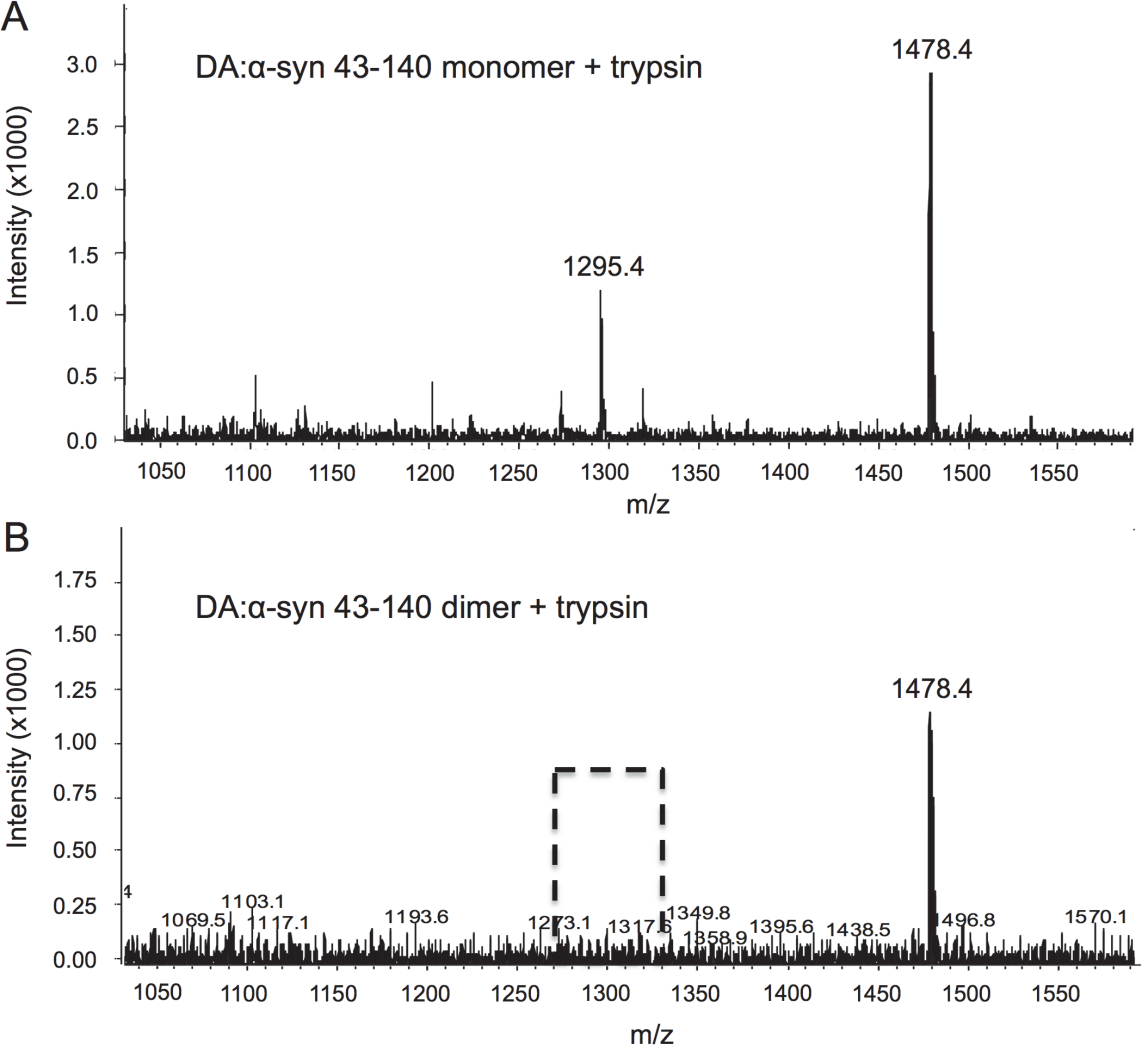
Differences between trypsin treatment of DA:α-syn 43–140 monomer and dimer. **A**. Mass spectrum of the DA:α-syn 43–140 monomer after overnight digestion at 37°C by trypsin. A major mass at 1478.4 Da was observed corresponding to residues 81 to 95, while a mass at 1295.4 Da is consistent with a fragment between residues 46 to 58. **B**. Trypsin cleaves the DA:α-syn 43–140 dimer into a single detectable fragment. Mass spectrum of the trypsin digest of the DA:α-syn 43–140 dimer reveals a single peak of mass 1478.4 Da, and the absence (broken line box) of the 1295.4 Da fragment formed by the DA:α-syn 43–140 monomer digest. This suggests this fragment is potentially modified upon interaction with DA.

To elucidate potential modifications/adducts mediated by DA the DA:α-syn 43–140 monomer and dimer proteins were trypsin digested and analysed by mass spectrometry. Trypsin digested DA:α-syn 43–140 monomer gave a mass at 1478.4 Da (corresponding to a fragment encompassing residues 81 to 95, Fig. 8A). Another fragment was observed at m/z 1295.4 Da corresponding to residues 46 to 58. The smaller fragments at 247 Da, 146 Da and 559 Da were indistinguishable from the masses of the buffer salts and DA and could not be unequivocally identified. The mass spectrum of trypsin digested DA:α-syn 43–140 dimer showed a major mass at 1478.4 Da corresponding to residues 81 to 95. However, the 46 to 58 fragment (mass of 1295.4 Da) present in the DA:43–140 monomer digest was absent (Fig. 8B). The absence of this species suggests this fragment contains residues that have been modified in the formation of the dimer. However, we did not detect the appearance of a corresponding new mass. The modification could have resulted in inhibition/alteration of the trypsin cleavage site or the creation of a much larger, but undetected fragment, or it may not have ionised.

### His50, Glu83, and the NAC region are not necessary for DA-mediated oligomerisation, but the NAC region is required for formation of discrete oligomeric species

**Fig 9:**
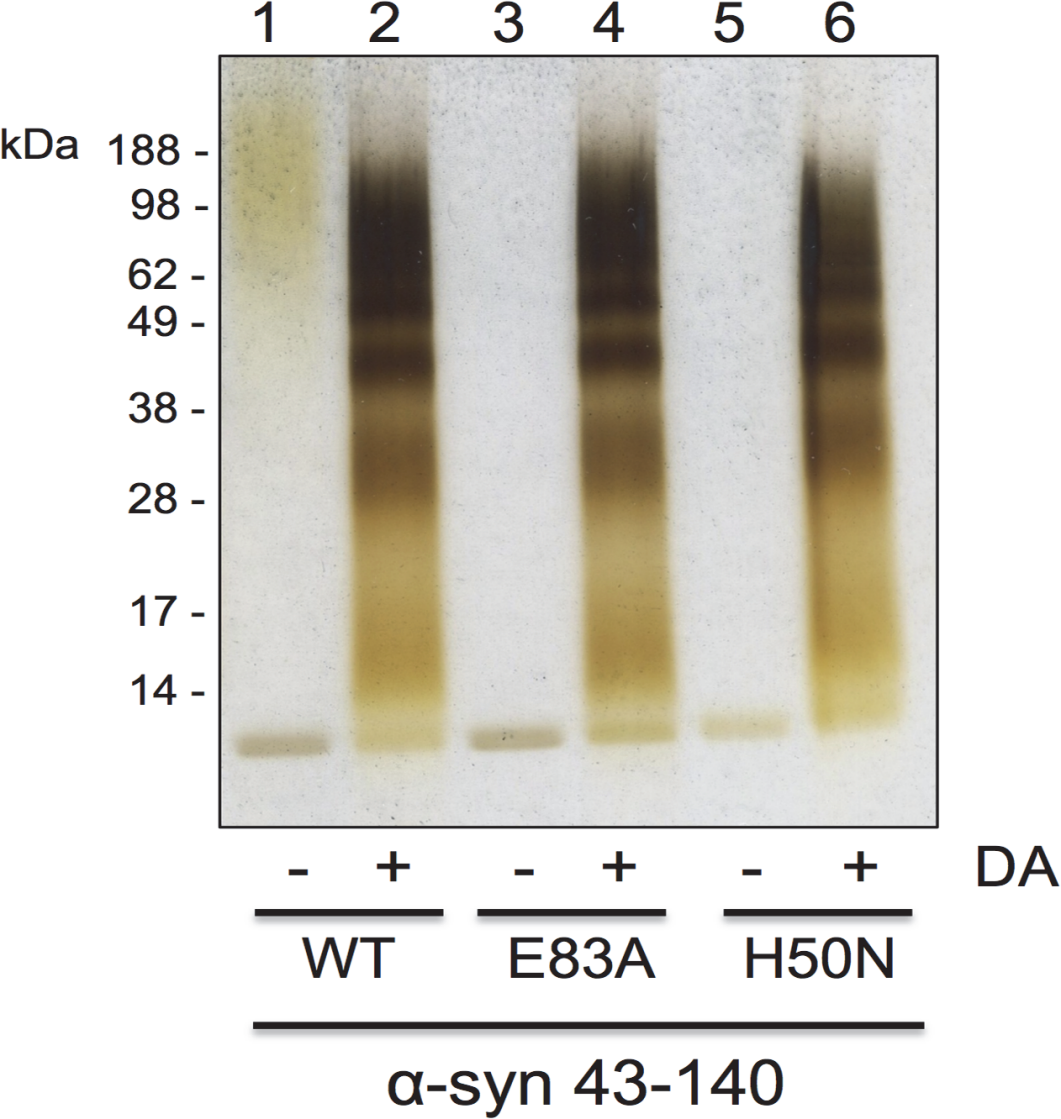
α-syn point mutants form oligomers. Silver stain SDS-PAGE gel of soluble fraction of mutant α-syn reacted with DA. Lane 1: α-syn 43–140, Lane 2: α-syn 43–140 + DA (1:10), Lane 3: α-syn 43–140 E83A, Lane 4: α-syn 43–140 E83A + DA (1:10), Lane 5: α-syn 43–140 H50N, Lane 6: α-syn 43–140 H50N+ DA (1:10)

The formation of DA:α-syn soluble oligomers is presumed to require an oxidative reaction [10, 12, 17, 19], since the methionine residues in α-syn are oxidised by DA and methionine-less α-syn mutant does not form soluble oligomers. [17] Histidine is another residue that is prone to oxidative modification [48, 49] and α-syn has a single histidine residue at position 50 (H50) which is in the proposed 43–60 interface region. H50 was mutated to asparagine (H50N) and tested for its ability to undergo DA-mediated oligomerisation. α-syn 43–140:H50N formed DA-mediated soluble oligomers similar to wildtype α-syn 43–140 (Fig. 9), therefore, His50 is not critical for DA-mediated oligomerisation. A previous report had implicated E83 to be necessary for DA-mediated α-syn fibrillisation. [28] We tested the role of E83 in our system by incubating the α-syn 43–140 E83A mutant with DA. The α-syn 43–140 E83A was capable of forming DA-mediated soluble oligomers similar to those formed by α-syn 43–140 (Fig. 9) indicating E83 is not necessary for DA-mediated oligomerisation of α-syn 43–140.

**Fig 10:**
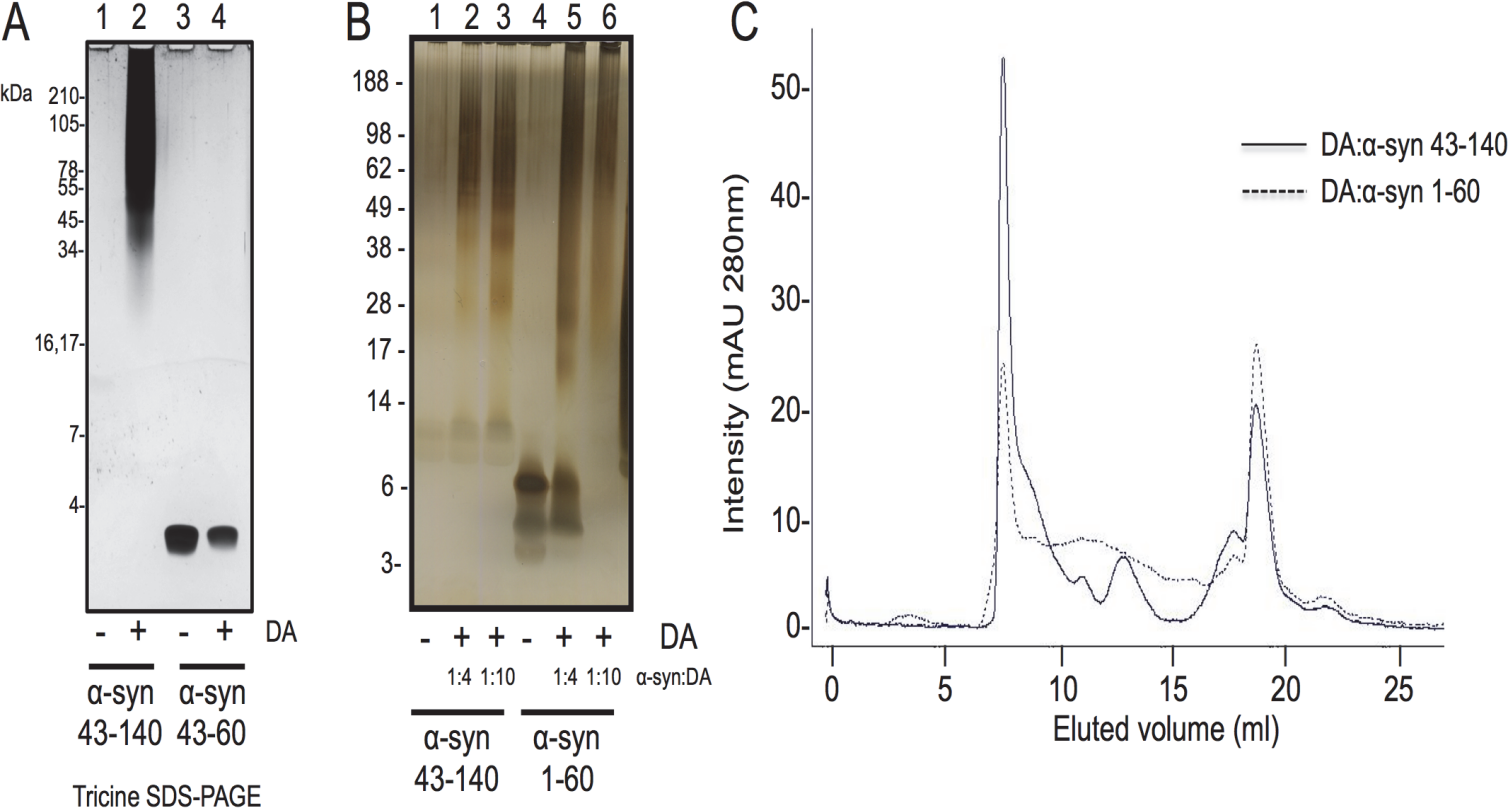
Truncation mutants of α-syn form oligomers after treatment with DA. Truncated α-syn and mutants were treated with DA and the soluble fraction subjected to SDS-PAGE and visualised by silver staining **A**. Tricine SDS-PAGE. Lane 1: α-syn 43–140, Lane 2: α-syn 43–140 + DA, Lane 3: α-syn 43–60, Lane 4: α-syn 43–60 + DA. **B**. Lane 1: α-syn 43–140, Lane 2: α-syn 43–140 + DA (1:4), Lane 3: α-syn 43–140 + DA (1:10), Lane 4: α-syn 1–60, Lane 5: α-syn 1–60 + DA (1:4), Lane 6: α-syn 1–60 + DA (1:10). **C**. SEC of the α-syn 43–140 and α-syn 1–60 DA induced oligomers. 200 μM α-syn was incubated with 2 mM DA for 7 days. The reaction was centrifuged at 100,000 rpm, 1 hour, 4°C and then analysed on a Superdex 200 10/300GL column using 10 mM sodium phosphate pH 7.5 buffer with a flow rate of 0.5 mL/min. Proteins were detected at A280nm. The solid line represents α-syn 43–140 and broken line α-syn 1–60.

The NMR data acquired on DA:α-syn 43–140 suggests that residues in the region 43–60 are potentially involved in the formation of the DA:α-syn soluble dimers. To examine this, we tested whether DA could induce oligomerisation of a peptide that spans residues 43–60 of α-syn. We analysed the reaction by Tricine SDS-PAGE which is better suited for separating small molecular peptides. [50] Incubation of α-syn 43–60 with DA did not induce soluble oligomers, as detected by Tricine SDS-PAGE and silver staining (Fig. 10A), suggesting sequences outside, or in addition to, residues 43–60 are required for oligomer formation. We also analysed the reaction by both MALDI and electrospray mass spectrometry and the only species detected was the monomeric peptides, and no higher molecular weight species were detected (data not shown). To test if the NAC fragment was necessary for DA-mediated oligomerisation, we examined the behaviour of α-syn 1–60 which represents a NAC-deleted species. α-syn 1–60 formed soluble oligomers after treatment with DA and in a dose dependent manner. However, this species did not migrate as a discrete band typically seen for α-syn 43–140 (Fig. 10B). We used SEC to determine how the DA:α-syn 1–60 species migrated in solution as compared to α-syn 43–140. Consistent with the SDS-PAGE data the DA:α-syn 1–60 and DA:α-syn 43–140 had distinct profiles with DA:α-syn 43–140 displaying multiple distinct peaks while DA:α-syn 1–60 showed only a single major peak and lacked distinct species in the region between 10–15 mL in the elution profile. (Fig. 10C).

**Fig 11:**
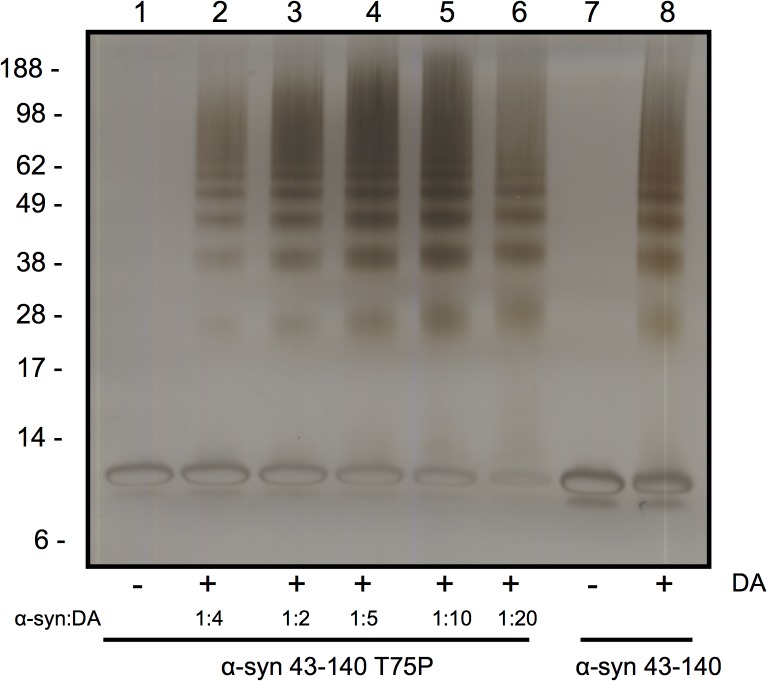
The NAC region is not required for DA mediated oligomerisation. The NAC fibrillsation mutant T75P did not alter DA mediated oligomerisation of α-syn 43–140. α-syn 43–140 T75P was incubate with different ratios of DA from 1:1 to 1:20 and analysed by SDS-PAGE and silver stain. Lane 1: α-syn 43–140 T75P, Lane 2: α-syn 43–140 T75P + DA (1:1), Lane 3: α-syn 43–140 T75P + DA (1:2), Lane 4: α-syn 43–140 T75P + DA (1:5), Lane 5: α-syn 43–140 T75P + DA (1:10), Lane 6: α-syn 43–140 T75P + DA (1:20), Lane 7: α-syn 43–140, Lane 8: α-syn 43–140 + DA (1:10)

The hydrophobic NAC region (residues 60–95) abuts 43–60 and is required for α-syn fibrillisation. A proline scan of this region identified residues that inhibited fibril formation, such as threonine 75. [51] To examine the role of the NAC region in DA-mediated oligomerisation we tested the α-syn 43–140 T75P mutant and found it formed DA-mediated SDS-stable soluble oligomers in a dose-dependent manner (Fig. 11). Therefore, while the NAC region is not required for oligomerisation, it is necessary for ensuring discrete oligomeric species are generated and there are potentially multiple mechanisms for oligomerisation.

## DISCUSSION

The N-terminally truncated 43–140 α-syn dimer was chosen for structural characterisation, over the C-terminal truncation or full-length α-syn, as α-syn 43–140 formed DA-mediated soluble oligomers with a similar oligomeric pattern to full length α-syn but significantly simplified the NMR analysis by reducing the number of sequence repeats to minimize chemical shift redundancy in the spectra. Moreover, the ESI-IMS-MS analysis of 43–140 α-syn showed it behaved in a similar manner to full-length α-syn by occupying both extended and compact conformations under neutral conditions. In the presence of DA the highly extended state monomeric state bound three DA ligands without the need to oxidise the Met residues. These similarities between 43–140 and full-length α-syn support the utility of using the 43–140 construct to investigate which regions of α-syn are required for the formation of the DA-mediated oligomers. In this study we compared the monomeric and dimeric species in order to understand the mechanism by which DA mediates α-syn oligomerisation.

CD and NMR spectra showed the DA:α-syn 43–140 dimer adopted a largely unstructured conformation in solution. The ^1^H, ^15^N HSQC spectrum showed amide resonances with limited chemical shift dispersion, characteristic of an IDP [52], while CD spectra had a single minimum of negative ellipticity at 200 nm and negligible ellipticity at 220 nm, again indicative of an IDP [53] The majority of amide resonances in the ^1^H,^15^N HSQC of α-syn 43–140 dimer were unshifted compared with those in the monomer, indicating that there is not a gross conformational change when compared to the monomer. Amide resonances of T43, V48, V49, H50, G51, T59, K60, E114, M116, V118, E126, M127, and S129 for the α-syn 43–140 dimer, are shifted or broadened compared to those in the monomer. These changes are centred around the N-terminus and the M127 residue at the C-terminus and indicates changes in chemical environment surrounding specific residues upon interaction with DA. Furthermore, the similarity in the ^1^H,^15^N-HSQC spectra between the dimer and monomer α-syn 43–140 suggests that, only a limited number of residues are modified by DA.

While CD, NMR and mass spectrometry data identified very few differences between the DA:α-syn 43–140 dimer and monomer, the dimer displayed altered behaviour in a membrane-mimetic environment. The presence of SDS caused α-syn 43–140 monomer to adopt α-helical structures and similar behaviour has been observed for full length α-syn. [45, 54–56] It is interesting to note that this occurs even without the first 42 residues of α-syn, which are a key part of the α-helical region. [34] Presumably, the stretch between residues 43 to 60, just after the purported break in the α-helical region, is sufficient for the initiation of α-helical formation in the presence of SDS [56] and consistent with our observation that residues 58–60 are the most affected, apart from those that are close to methionine, by DA. We have shown that lipids inhibit the formation of DA:α-syn oligomers, while the DA:α-syn oligomer cannot interact with lipid vesicles or cause membrane permeability. [23] In the current study, the observation that DA:α-syn 43–140 dimer did not form α-helices in the presence of SDS, is consistent with residues 43 to 60 being modified in dimer formation. Support for residues 43 to 60 being involved in the formation of soluble oligomers came from the trypsin digestion analysis of the DA:α-syn 43–140 dimer. Mass spectrometry of the trypsin generated fragments showed that the fragment corresponding to residues 46 to 58 was not detected following digestion of DA:α-syn 43–140 dimer (again consistent with the 58–60 region). This indicated potential modification in this region en route to the formation of the dimer. However, no corresponding new mass fragments were detected and therefore the identity of the modification remains unresolved. The importance of residues 43–60 was highlighted by α-syn 1–60 being capable of forming SDS-stable soluble oligomers in a dose dependent manner, with an oligomeric profile similar to that of DA:α-syn 43–140 and full length DA:α-syn. The α-syn 1–60 construct lacks the NAC region (61–95) and its ability to form oligomers confirms the NAC region is not necessary for playing a significant role in oligomer formation.

Our studies expand upon the NMR and biophysical analysis of full length α-syn modified by DA quinones [16] which showed the modified α-syn retained its unfolded conformation. Of particular relevance to our study is that only a small number of α-syn molecules were covalently modified by the DA quinones. [16] This may explain our inability to detect the covalent modification keeping the α-syn dimer together. The lack of major differences being observed in the NMR and CD spectra on treatment of α-syn 43–140 with DA suggests the interface of dimerisation or any structured core region is small. Only a few residues between 43 to 60 are involved (T43, V48, V49, H50, G51, T59, K60), potentially via lysine residue modifications, while the rest of the protein remains disordered in solution. Interestingly, the N-terminal region of α-syn, between residues 1–60, was found to be important for the binding of exifone, an inhibitor of α-syn aggregation adding weight that this region modulates α-syn aggregation. [57] Our NMR analysis implicated a role for the C2 of His50, however, the α-syn 43–140 H50N mutant could still form oligomers in the presence of DA. This suggests that the histidine residue is not solely responsible for the formation of oligomers and that other residues in α-syn 43–140 may also be involved, or that other residues may assume the role of His50 in the H50N mutant. While α-syn E83A was proposed to abolish the ability of DA to inhibit α-syn fibrillisation. [28], in our hands α-syn 43–140 E83A could form DA-mediated soluble oligomers. This supports the model where the pathway towards formation of DA-mediated soluble oligomers is distinct to that of α-syn fibrillisation. While the T75P mutant in the NAC region was also ineffective in altering DA-mediated α-syn oligomerisation and it may point to multiple mechanisms for dimer formation.

This study has provided novel and important data on the structural characteristics of the DA:α-syn oligomer, and the contribution of distinct domains and residues to their formation. We have narrowed the interface to a small region and while the exact residues involved and the nature of the interaction with DA / DA-products remains unresolved, our new data further defines the mechanism by which DA mediates the formation of these SDS-stable soluble DA:α-syn oligomers.

## Supporting Information

S1 Fig Oligomerisation of α-syn treated with pre-oxidised DA. Silver stained SDS-PAGE gel of the different α-syn proteins treated with either freshly prepared or pre-oxidised DA. No DA refers to the proteins minus the addition DA. Fresh DA refers to DA (200uM) that has been added to the α-syn soon after the DA has been dissolved, as per the other incubations performed in this study, i.e. Fig. 1B. Oxidised DA supernatant is the supernatant fraction of the 28 day reacted DA; Oxidised DA is the pellet fraction of the 28 day reacted DA that was then resuspended into water. DA ctl, oxDA supernatant and oxDA pellet is the reaction with just the specific DA species indicated but minus α-syn, in order to show what staining is due to the DA. The α-syn to DA ratio was 1:10.
(TIF)
