# Supplementary figures and images for: The N-Terminal Residues 43 to 60 Form the Interface for Dopamine Mediated α-Synuclein Dimerisation
Source: PLoS One. 2015 Feb 13;10(2):e0116497. doi: 10.1371/journal.pone.0116497 (PMC4332483; doi:10.1371/journal.pone.0116497)

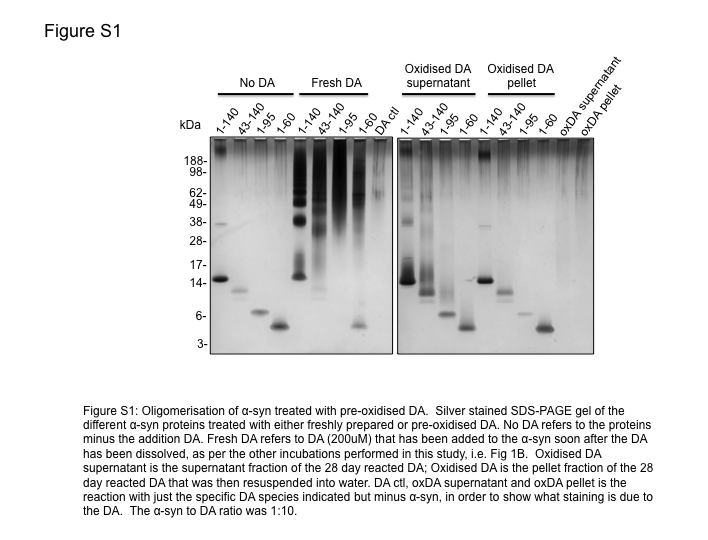

Supplement: S1 Fig — Silver stained SDS-PAGE gel of the different α-syn proteins treated with either freshly prepared or pre-oxidised DA. No DA refers to the proteins minus the addition DA. Fresh DA refers to DA (200uM) that has been added to the α-syn soon after the DA has been dissolved, as per the other incubations performed in this study, i.e. Fig. 1B. Oxidised DA supernatant is the supernatant fraction of the 28 day reacted DA; Oxidised DA is the pellet fraction of the 28 day reacted DA that was then resuspended into water. DA ctl, oxDA supernatant and oxDA pellet is the reaction with just the specific DA species indicated but minus α-syn, in order to show what staining is due to the DA. The α-syn to DA ratio was 1:10. (TIF) [file pone.0116497.s001.tif]
